# Does Social Isolation and Low Societal Participation Predict Disability Pension? A Population Based Study

**DOI:** 10.1371/journal.pone.0080655

**Published:** 2013-11-06

**Authors:** Klas Gustafsson, Gunnar Aronsson, Staffan Marklund, Anders Wikman, Birgitta Floderus

**Affiliations:** 1 Division of Insurance Medicine, Department of Clinical Neuroscience, Karolinska Institutet, Stockholm, Sweden; 2 Department of Psychology, Stockholm University, Stockholm, Sweden; Marienhospital Herne - University of Bochum, Germany

## Abstract

**Purpose:**

The aim was to examine the potential influence of social isolation and low societal participation on the future risk of receiving disability pension among individuals in Sweden. A specific aim was to describe differences depending on disability pension diagnoses, and how the results were modified by sex and age.

**Method:**

The study comprised representative samples of Swedish women and men, who had been interviewed in any of the annual Swedish Surveys of Living Conditions between 1990 and 2007. Information on disability pension and diagnoses was added from the Swedish Social Insurance Agency’s database (1991-2011). The mean number of years of follow-up for the 53920 women and men was twelve years (SD 5.5), and the study base was restricted to the ages 20 to 64 years of age. The predictors were related to disability pension by Cox’s proportional hazards regression.

**Results:**

Social isolation and low societal participation were associated with future disability pension also after control for age, year of interview, socio demographic conditions and self reported longstanding illness. Lone individuals were at increased risk of disability pension, and the effect of living without children was modified by sex and age. An increase in risk was particularly noticeable among younger women who reported that they had sparse contacts with others, and no close friend. Both women and men who reported that they did not participate in political discussions and who could not appeal on a decision by a public authority were also at increased risk. The effects of social isolation were mainly attributed to disability pension with mental diagnoses, and to younger individuals.

**Conclusions:**

The study suggests that social isolation and low societal participation are predictors of future disability pension. Social isolation and low societal participation increased particularly the risk of future disability pension in mental diagnoses among younger individuals.

## Introduction

To be integrated in society is an important aspect of an individual’s health and welfare [1,2], and poor integration is often accompanied by weak societal networks, e.g. being excluded from the labour market or participation in political life, and weak connections to other people with social isolation as an extreme situation [3,4]. Gallie et al. [1], have suggested that unemployment may lead to “erosion of social ties” which in turn could lead to social isolation. The chronology may also be the opposite, with the social conditions affecting the employment situation. Gallie et al. [1], defines three related spheres of “sociability”. The primary sphere includes the household and whether or not the individual is living alone. The secondary sphere comprise informal social networks such as how often people meet with friends, and how often they talk to neighbors. The tertiary sphere refers to societal participation including involvement in political life, ability to appeal to public decisions and membership in different forms of associations. These aspects of societal participation were introduced in the social indicators research in the late 1960s as components of the individuals political recourses [3,5]. Those who do not take part in political discussions and those who cannot appeal against a public authority decision are seen as less able to influence collective decision-making [3,5]. Similarly, those who are not members of any association, such as a political, religious or labour union organization are also seen as being in a weaker societal position as they are less integrated in organizational life [3,5].

Comparative studies have found differences between countries with respect to the dominance of these spheres [1,4]. Due to stronger family ties, the number of younger people in social isolation appears to be lower in southern compared to Northern Europe [1,4]. Although, there are many reasons for this, these countries also show comparatively low levels of disability pension (DP) [6]. In Sweden 44 % of the households consist of a single person, in Finland 41%, Denmark 39%, Italy 28 %, and Spain 18 % [7].

Few studies have explicitly examined social isolation and societal participation as predictors of DP, but some prospective studies have indicated an association between risk of DP and lack of social support [8,9], which may be seen as an aspect of social isolation. A Finnish study found that the association between low social support from superiors at work and receiving DP was mediated by the individual’s health status [9]. However, a Norwegian prospective study indicated that the association between private life support and DP was weak [10]. Similarly weak associations were found between general social support and future DP in a prospective cohort study among employees in Denmark [11]. 

**Figure 1:**
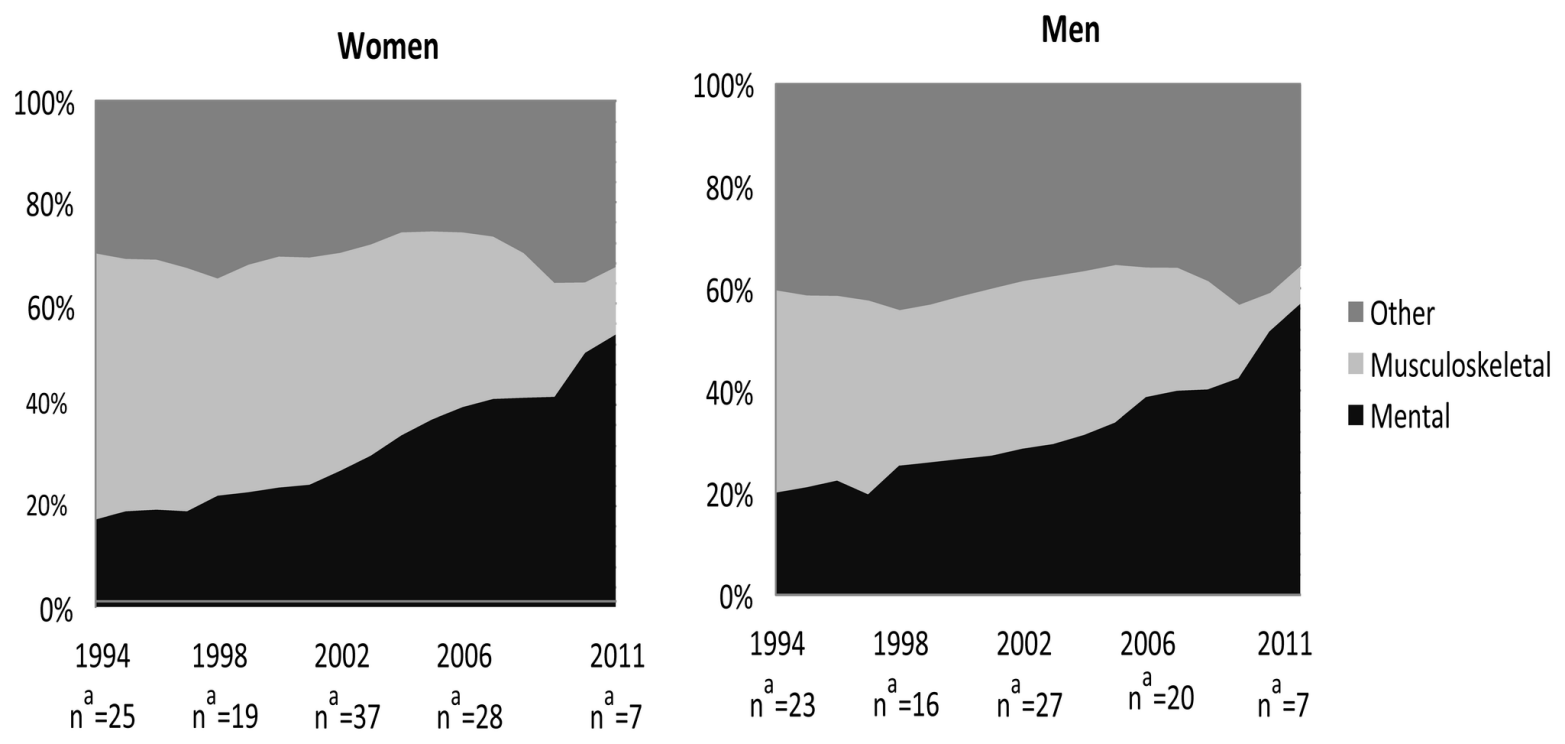
The total number of new cases of DP in 1000s. ^a^ The proportion of mental, musculoskeletal, and other diagnoses among all newly-granted disability pensions in 1994 to 2011, in Swedish men and women (based on data from Swedish Social Insurance Agency, 2012).

In recent years, permanent exit from the work force on health grounds has increased among younger people in all the OECD countries [12]. In 2011, almost 401 000 individuals in Sweden had been granted permanent or temporary DP [13]. This means that approximately 7% of the Swedish population aged 19 to 64 entirely or partially had left the labor market [13]. Before 2004, musculoskeletal diagnoses dominated among newly-granted cases of DP in Sweden [13] (Figure 1). Since 2005, mental diagnoses have been the most common diagnostic group in DP in both women and men [13]. Mental diagnoses accounted for 54% and 57% of all newly granted DPs in women and men aged 19-64, respectively [13]. In the youngest age group (19-29 years) the shares were even higher (74% and 77%) [24].

The trend of increasing shares of young people on DP due to mental disorders has also been reported from other Nordic countries [14], Western Europe [12] and other industrialized countries [6,15]. Norwegian researchers have shown that DP due to mental diagnoses was granted on average nine years earlier than DP with musculoskeletal diagnoses, and that DP with mental diagnoses caused the highest number of lost working years compared with all other DP diagnoses [16]. Furthermore, the study found that depressive illness may contribute to DP more often than is officially reported [17] and that 25 % of people who were recently granted a DP with mental diagnosis had never sought treatment for mental problems [18,19]. There is also evidence from other studies that mental diagnoses are often under-recognized and untreated in the primary care setting [20-23]. 

The present study is an expansion of our previous studies on predictors of DP [24,25], including work status and family status in young women. Here, we wanted to extend the focal point to social isolation, and societal participation among both men and women at different ages. The aim was to examine prospectively the potential influence of social isolation and low societal participation on the risk of going on DP. Specific aims were to describe differences depending on the DP diagnosis, i.e. mental and musculoskeletal diagnoses, and how the results were modified by sex and age. 

## Methods

### Study Group

The study comprised 53 920 men and women, 20 to 64 years old during follow-up (born between 1928 and 1987), who were interviewed by Statistics Sweden between 1990 and 2007 within the Swedish Surveys of Living Conditions (SSLC), covering a broad range of living conditions [26,27]. The annual surveys were based on year-specific random samples of the Swedish population and conducted as face-to-face interviews in the years 1991 to 2005 and as telephone interviews in the years 2006 and 2007. The annual response rates (1990-2007) varied between 80 and 76 %. If an individual happened to be included in the sample of more than one year, data from the first year was used in this study. The study group was further linked to two other population registers the Longitudinal Database for health insurance and labor market studies (LISA) (1990-2011) and the Swedish Social Insurance Agency’s database Micro Data for Analysis of Social Insurance (MiDAS) (1991-2011).

Men and women who had obtained a DP prior to being interviewed (n=4128) were excluded from the study. Of the 53 920 remaining individuals, 5724 (10.6 %) were granted DP within the follow-up period that covered the years 1991-2011. Follow-up started one year after the interview and ended in 2011. Thus, the follow-up time varied between one year and 20 years and was highly dependent on age and when the interview was conducted. Table 1 shows the characteristics of the study group, including age at interview, number of person years at risk of DP, and mean age when DP was granted stratified by sex and status of DP. 

**Table 1:** Disability pension (DP) 1991-2011, person years at risk and mean age at DP. Women and men interviewed in 1990-2007, n=53920.

	Total		No disability pension		Disability pension			Age at	
	n=53920		n=48196		n=5724			Disability pension	
	Men	Women	Men	Women	Men	Women		Men	Women
	n	n	n	n	n	n		Mean, SD	Mean, SD
Total	27114	26806	24775	23421	2339	3385			
Age									
20-39 year	14030	13852	13458	12772	572	1080			
40-64 year	13084	12954	11317	10649	1767	2305			
DP diagnoses									
Mental^a^					547	913		47.7(10.3)	47.9(10.0)
Musculoskeletal^b^					771	1435		54.7(8.0)	52.6(8.5)
Other specific^c^					935	920		54.9(8.4)	52.4(9.1)
Unspecified^d^					86	117		56.7(6.8)	54.8(9.0)
All DP^e^					2339	3385		53.2(9.2)	51.4(9.4)
Person years at risk	Years	Years	Years	Years	Years	Years			
Total n=683819	346084	337735	328759	312745	17325	24990			
Mean number									
of years (Mean, SD)	12.8(5.5)	12.6(5.6)	13.3(5.3)	13.4(5.3)	7.4(4.5)	7.4(4.4)			

^a^ Mental DP-diagnoses (ICD-10, F00-F99), granted 1994-2011.

^b^ Musculoskeletal DP-diagnoses (ICD-10, M00-M99), granted 1994-2011.

^c^ Other specific DP-diagnoses (ICD-10, A-E, G-L, N-Z), granted 1994-2011.

^d^ Unspecified DP-diagnoses, granted 1991-1993.

^e^ All DP, including unspecified diagnoses, granted 1991-2011.

### Outcome Variables

Three categories of DP were used: All DP-cases (granted 1991-2011) (n=5724), DP with mental diagnoses (ICD-10, F00-F99) (granted 1994-2011) (n=1460) and, musculoskeletal diagnoses (ICD-10, M00-M99) (granted 1994-2011) (n=2206) (Table 1). The DP could be either full time or part time (25, 50, 75%) but in this study no distinction was made concerning full-time or part time DP. The data were obtained from the MiDAS database. 

The category “All DP” included all diagnostic groups [28] as well as 203 cases without a specified diagnosis. Most of these unspecified cases received a DP in the years 1991 and 1992 and were mainly caused by the fact that before 1992, individuals 60-64 year of age could receive a DP primarily due to labor market reasons, with more relaxed criteria concerning ill health and reduced work capacity.

### Exposure variables

Inspired by the mentioned study by Gallie et al [1] lack of “sociability” at the primary and secondary sphere involving family and friends were in the present study defined as *social isolation*. The third sphere in Gallies classification was here defined as low *societal participation* and included membership in organizations as well as participation in political discussions and the ability to appeal to decisions made by public authorities. The concept societal participation reflects the individuals’ perception about their ability to make themselves heard. Three aspects of competence and engagement in societal issues have been used here. The first aspect, participation in political discussion, covers whether one regards oneself as a political person. About 25% of the Swedish population claim that they do not take part in political discussions [29]. The second, ability to appeal, relates to the individuals experience and perception of public authorities. Less than 6% report that they cannot appeal to decisions made by a government authority [29]. The last, membership in a work related organization such as trade union, agricultural organization, employer’s organization, focuses on integration in organizational life. In Sweden organizational membership has generally been high but has declined slightly over time and presently about 20% are not members of any organization [29]. All items measuring the exposure variables have been developed within the SSLC and have been used in many studies [26,30,31].

#### Social isolation

The following three variables were used to measure *social isolation*:

- *Family* *status*: cohabiting with children (reference); cohabiting without children; lone with children; and lone without children.
- *Social* *contacts*: “In general how often do you meet with friends, acquaintances or relatives? Do not include current neighbors or workmates”. The response scale was: several times a week (reference); sometime per week; sometime per month; and more seldom.
- *Having close friends*: “Do you have one or more really close friend with whom you can get in contact and discuss all sorts of things? Do not include members of your family or your household. The response choices were: yes (reference) and no.

#### Low societal participation

The following three variables were included to estimate low societal participation because they may indicate the extent to which the individual did take part in organizational and societal activities.

- *Participation* *in* *political* *discussions*: “How do you usually do if you are together with people and the discussion comes to politics?” The response choices were: I usually take part in discussions and give my opinion when it comes to politics (reference); It happens sometimes but not so often that I take part in discussions and give my opinion; I usually listen, but I do not take part in political discussions; I usually do not listen and I do not take part in political discussions.
- *Ability* *to* *appeal*: “Could you write a letter and appeal a decision made by a government agency?”. The response choices were: I can write a letter and appeal a decision made by a government agency (reference); I cannot appeal a decision made by a government agency, but I know a person who can help; I cannot appeal a decision made by a government agency and I do not know a person who can help, but I know where to turn for help; I cannot appeal a decision made by a government agency and I do not know a person who can help, and I do not know where to turn for help. The last two response choices were reclassified into one category.
- *Membership* *in* *a* *work* *related* *association*: “Are you a member of any trade union, agricultural organization, employer’s organization or some such body?” The response choices were: yes (reference) and no.

### Potential confounders

#### Socio demographic conditions

Three variables indicating socio demographic conditions were selected as potential confounding factors. These were country of birth, work status, and educational level. Previous studies have found that being foreign born increases the risk of DP [25,32]. Individuals whose work status involves unemployment or a peripheral relation to the labor market are also at a higher risk for DP [33,34]. A number of studies have also shown that low educational level is related to DP [33,35]. The measurements of country of birth and work status at interview originate from the SSLC database and educational level at interview was obtained from the LISA database:

- *Country* *of* *birth*: born in Sweden with Swedish-born parents (reference); born in Sweden, with one or both parents foreign born; and foreign born.
- *Work* *status*: Being employed or being a student (reference); not employed: job seeking, being a homemaker or not possible to classify (those whose employment status or occupation could not be established, about 1.4 % of all individuals).
- *Education*: less than secondary education (< 9 years of education); some or all secondary education completed (10-12 years); and post-secondary education (>12 years) (reference).

#### Health and longstanding illness

Since poor health could contribute to social isolation and low societal participation and is a prerequisite for being granted DP, the individuals’ health status was controlled for. It should be noted however that self rated health (SRH) and self-reported longstanding illness were assessed at the same point in time as social isolation and societal participation. Items measuring SRH and self reported longstanding illness were obtained from the SSLC database [36,37].

- Self-rated health (SRH) was measured using the question: “How do you rate your general health condition?” The five-digit scale was dichotomized into good (very good/good) reference; and poor (in-between/bad/very bad).
- *Self* *reported* *longstanding* *illness* was measured by the open-ended questions: “Do you have any chronic or longterm illness or health problem?” The answers were followed up by the trained interviewers at Statistics Sweden to provide a solid basis for coding according to the WHO International Classification of Disease, 8^th^ revision (ICD-8). The summary coding: yes and no (reference) were used in this study.

### Statistical analyses

The selected participants from the annual SSLC surveys, from 1990 to 2007, were consecutively added to the cohort and the follow-up period for each sub-cohort started the year after the interview (January 1, 1991-2011). The follow-up period for the participants ended on November 30, 2011, or the year they reached 64 years of age, went on DP, emigrated or died, whichever came first (1991-2010). Hazard ratios (HRs) of being granted a DP, with 95% confidence intervals (CI) were estimated by Cox’s proportional hazards regression analysis. All statistical analyses were conducted with SAS, version 9.2., statistical software (SAS Institute, Inc., Cary, North Carolina) using the PHREG procedure.

All analyses were stratified on sex and age: men and, women 20-39 years of age, men and, women 40-64 years of age. Median age at DP was 41.0 years (mean 40.6 years) for people aged 20 to 39 at interview, while people aged 40 to 64 years at interview had a median DP age of 58.0 years (mean, 56.8 years) when they were granted a DP. 

The statistical analyses were conducted in three steps. First, the importance of socio demographic conditions, and self-reported health were related to the risk of DP, adjusting for age (one - year intervals) and year of interview (Table 2). Secondly, social isolation and low societal participation were related to risk of DP, adjusting for a) age at interview (one-year intervals) and year of interview, b) corresponding to a) with socio demographic conditions and self-reported longstanding illness added to the model (Table 3). Thirdly, DP with mental diagnoses and with musculoskeletal diagnoses were studied separately according to the procedure described above, the results according to b) were reported (Table 4, 5).

**Table 2:** Socio-demographic conditions and self reported health related to risk of DP^**a**^, controlling for age at interview and year of interview.

	Ages 20-39								Ages 40-64							
	Men (n=14030)				Women (n=13852)				Men (n=13084)				Women (n=12954)			
	P^b^	HR^c^	CI		P^b^	HR^c^	CI		P^b^	HR^c^	CI		P^b^	HR^c^	CI	
**Socio-deomgraphic conditions**																
**Country of birth**																
Born in Sweden with																
Swedish-born parents	79	**1**			77	**1**			85	**1**			84	**1**		
Born in Sweden, with one																
or both parents foreign born	10	1.13	0.84	1.51	10	**1.39**	1.14	1.69	4	0.81	0.60	1.11	4	0.98	0.78	1.24
Foreign born	11	**2.35**	1.91	2.89	13	**1.77**	1.51	2.06	11	**1.78**	1.57	2.03	12	**1.45**	1.29	1.63
**Education**																
Post secondary education	27	**1**			32	**1**			27	**1**			31	**1**		
Secondary education	59	**2.11**	1.63	2.72	56	**2.00**	1.68	2.37	42	**1.79**	1.56	2.06	44	**1.57**	1.41	1.75
Less than secondary education	14	**4.04**	3.06	5.33	12	**4.41**	3.65	5.32	30	**2.33**	2.02	2.68	25	**1.67**	1.49	1.88
**Work status**																
Employed/Students	95	**1**			90	**1**			93	**1**			90	**1**		
Homemakers, jobseeking																
unclassified	5	**4.05**	3.21	5.11	10	**2.81**	2.42	3.26	7	**1.81**	1.55	2.12	10	**1.24**	1.08	1.41
**Self reported health**																
**Self rated health**																
Good	72	**1**			70	**1**			60	**1**			57	**1**		
Poor	28	**5.23**	4.43	6.19	30	**4.68**	4.15	5.28	40	**4.14**	3.76	4.55	43	**3.96**	3.65	4.30
**Longstanding illness**																
No	88	**1**			85	**1**			81	**1**			79	**1**		
Yes	12	**3.42**	2.90	4.04	15	**3.53**	3.13	3.98	19	**3.27**	2.97	3.61	21	**3.21**	2.94	3.50

^a^ All incident cases of DP, including unspecified DP-diagnosis (n=5724).

^b^ Prevalence (P) of the exposure categories (%).

^c^ Hazard ratio (HR) and 95% confidence interval (CI), adjusted for age at interview, and year of interview.

**Table 3:** Social isolation, and societal participation related to risk of DP^**a**^, controlling for age at interview, year of interview, socio-demographic conditions and long-standing illness at interview.

	Ages 20-39														Ages 40-64												
	Men (n=14030)							Women (n=13852)							Men (n=13084)							Women (n=12954)					
	P^b^	n^c^						P^b^	n^c^						P^b^	n^c^						P^b^	n^c^				
	%	572	HR^d^	HR^e^	CI			%	1080	HR^d^	HR^e^	CI			%	1767	HR^d^	HR^e^	CI			%	2305	HR^d^	HR^e^	CI	
**Social isolation**																											
**Family status**																											
Cohabiting, with children	34	225	**1**	**1**				46	584	**1**	**1**				40	577	**1**	**1**				32	605	**1**	**1**		
Cohabiting, no children	19	79	1.04	1.07	0.82	1.40		20	134	0.82	0.90	0.74	1.10		40	742	**1.26**	**1.22**	1.07	1.38		45	1113	**1.66**	**1.49**	1.33	1.68
Lone, with children	1	15	**1.92**	1.66	0.96	2.85		7	161	**1.86**	**1.52**	1.27	1.82		2	35	1.22	1.14	0.81	1.61		7	186	**1.65**	**1.53**	1.30	1.81
Lone, no children	46	253	**1.58**	**1.52**	1.24	1.85		27	201	1.14	**1.28**	1.07	1.54		18	413	**1.76**	**1.58**	1.39	1.80		16	401	**1.73**	**1.57**	1.36	1.80
**How often contact**																											
**with others**																											
Several times a week	41	206	**1**	**1**				33	271	**1**	**1**				10	226	**1**	**1**				9	221	**1**	**1**		
Some times a week	34	187	**0.81**	0.91	0.74	1.12		39	379	0.94	1.01	0.86	1.18		31	512	**0.69**	**0.79**	0.67	0.92		32	736	0.89	0.91	0.78	1.06
Some times per month	18	122	0.84	0.95	0.75	1.21		21	283	1.09	1.14	0.96	1.36		40	640	**0.64**	**0.76**	0.65	0.89		42	909	**0.80**	**0.83**	0.71	0.96
More seldom	6	54	1.11	1.03	0.74	1.42		7	140	**1.79**	**1.46**	1.18	1.81		19	383	**0.82**	0.88	0.74	1.04		17	435	0.99	0.94	0.80	1.11
**Having close friends**																											
Yes	85	428	**1**	**1**				92	912	**1**	**1**				71	1209	**1**	**1**				87	1959	**1**	**1**		
No	15	133	**1.32**	1.19	0.97	1.45		8	161	**1.75**	**1.34**	1.13	1.60		29	531	1.05	1.01	0.91	1.12		13	334	**1.18**	1.06	0.95	1.20
**Societal participation**																											
**Participation in political**																											
**discussions**																											
Usually take part	49	249	**1**	**1**				43	378	**1**	**1**				48	762	**1**	**1**				41	873	**1**	**1**		
Sometimes take part	30	144	0.91	0.89	0.72	1.09		32	326	1.11	1.05	0.90	1.21		32	521	1.00	0.92	0.83	1.03		35	767	1.03	0.99	0.90	1.09
I usually listen, not take part	13	94	**1.51**	1.21	0.95	1.55		17	217	**1.53**	1.17	0.99	1.39		15	316	**1.32**	**1.14**	1.00	1.30		18	497	**1.28**	**1.12**	1.00	1.26
I do not listen or take part	8	69	**1.84**	**1.36**	1.03	1.79		9	151	**2.22**	**1.46**	1.20	1.79		5	141	**1.90**	**1.39**	1.16	1.67		5	154	**1.58**	**1.24**	1.04	1.49
**Ability to appeal**																											
Can appeal	76	372	**1**	**1**				68	701	**1**	**1**				77	1203	**1**	**1**				73	1554	**1**	**1**		
Can appeal with help	22	143	**1.43**	1.10	0.90	1.34		25	282	**1.23**	0.98	0.85	1.13		19	430	**1.51**	**1.14**	1.02	1.29		23	596	**1.25**	1.09	0.98	1.20
Can not appeal/no help	5	47	**2.35**	**1.76**	1.29	2.42		6	91	**1.91**	**1.28**	1.01	1.61		4	110	**2.00**	**1.39**	1.14	1.71		4	142	**1.75**	**1.39**	1.16	1.66
**Membership in a work**																											
**related association**																											
Yes	67	385	**1**	**1**				70	835	**1**	**1**				80	1441	**1**	**1**				83	2000	**1**	**1**		
No	33	186	**1.35**	**1.28**	1.06	1.53		30	242	0.96	**0.74**	0.64	0.86		20	323	0.97	0.95	0.84	1.07		17	302	**0.73**	**0.65**	0.57	0.74

^a^ All incident cases of DP, including unspecified DP-diagnosis (n=5724). ^b^ Prevalence (P) of the exposure categories (%). ^c^ Number of cases (n). ^d^ Hazard ratio (HR), adjusted for age at interview and year of interview. ^e^ Hazard ratio (HR) and 95% confidence interval (CI), adjusted for socio-demographic conditions (country of birth, education, work status), self-reported long-standing illness, age at interview and year of interview.

**Table 4:** Social isolation and risk of DP with mental and musculoskeletal diagnoses, respectively. Multivariate analyses.

	Ages 20-39									Ages 40-64							
	Men				Women					Men				Women			
	n^a^	HR^b^	CI		n^a^	HR^b^	CI			n^a^	HR^b^	CI		n^a^	HR^b^	CI	
**Mental diagnoses**	251				443					296				470			
**Social isolation**																	
**Family status**																	
Cohabiting, with children	70	**1**			203	**1**				101	**1**			129	**1**		
Cohabiting, no children	33	1.35	0.88	2.08	44	0.83	0.59	1.17		78	1.16	0.83	1.62	169	**1.68**	1.29	2.19
Lone, with children	5	1.43	0.52	3.92	82	**2.23**	1.72	2.90		8	1.47	0.71	3.02	61	**2.48**	1.83	3.37
Lone, no children	143	**2.53**	1.84	3.46	114	**2.02**	1.55	2.64		109	**2.85**	2.15	3.78	111	**2.77**	2.10	3.66
**How often contact**																	
**with others**																	
Severaltimes a week	96	**1**			118	**1**				55	**1**			45	**1**		
Sometimes a week	94	1.09	0.81	1.47	159	1.02	0.80	1.31		77	**0.51**	0.36	0.73	142	0.94	0.67	1.31
Sometimes per month	32	**0.63**	0.42	0.96	109	1.15	0.87	1.51		90	**0.50**	0.36	0.70	187	0.96	0.69	1.33
More seldom	27	1.40	0.89	2.20	51	**1.42**	1.01	2.01		72	0.82	0.57	1.16	95	1.25	0.87	1.79
**Having close friends**																	
Yes	183	**1**			362	**1**				198	**1**			395	**1**		
No	61	**1.35**	1.00	1.82	75	**1.70**	1.32	2.20		92	1.09	0.85	1.41	74	1.27	0.98	1.63
**Musculoskeletal diagnoses**	138				363					633				1072			
**Social isolation**																	
**Family status**																	
Cohabiting, with children	77	**1**			229	**1**				200	**1**			280	**1**		
Cohabiting, no children	18	0.78	0.45	1.33	47	0.85	0.61	1.18		297	**1.42**	1.15	1.76	558	**1.55**	1.31	1.83
Lone, with children	5	1.87	0.75	4.64	47	1.19	0.87	1.64		12	1.14	0.63	2.04	72	1.27	0.98	1.65
Lone, no children	38	0.74	0.48	1.15	40	0.72	0.49	1.04		124	**1.39**	1.11	1.76	162	**1.39**	1.12	1.71
**How often contact**																	
**with others**																	
Several times a week	43	**1**			82	**1**				63	**1**			98	**1**		
Sometimes a week	45	0.90	0.58	1.39	126	1.05	0.79	1.40		187	1.01	0.76	1.36	345	0.94	0.75	1.17
Sometimes per month	39	1.18	0.74	1.88	99	1.18	0.86	1.60		254	1.08	0.81	1.43	443	0.86	0.69	1.08
More seldom	10	0.64	0.29	1.39	55	**1.70**	1.18	2.44		127	1.04	0.76	1.41	185	0.86	0.67	1.10
**Having close friends**																	
Yes	105	**1**			308	**1**				435	**1**			914	**1**		
No	33	1.12	0.75	1.67	54	1.19	0.88	1.62		190	1.00	0.84	1.18	154	1.03	0.87	1.23

^a^ Number of cases (n).

^b^ Hazard ratio (HR) and 95% confidence interval (CI), adjusted for socio-demographic conditions, self-reported long-standing illness, age at interview, and year of interview.

**Table 5:** Societal participation and risk of DP with mental and musculoskeletal diagnoses, respectively. Multivariate analyses.

	Ages 20-39								Ages 40-64							
	Men				Women				Men				Women			
	n^a^	HR^b^	CI		n^a^	HR^b^	CI		n^a^	HR^b^	CI		n^a^	HR^b^	CI	
**Mental diagnoses**	251				443				296				470			
**Societal participation**																
**Participation in political**																
**discussions**																
Usually take part	97	**1**			160	**1**			129	**1**			196	**1**		
Sometimes take part	70	1.12	0.82	1.53	129	1.00	0.79	1.27	91	1.06	0.81	1.39	170	1.12	0.91	1.38
I usually listen, not take part	41	1.32	0.91	1.92	85	1.14	0.87	1.50	45	1.10	0.77	1.56	79	1.06	0.81	1.39
I do not listen or take part	34	**1.55**	1.03	2.31	62	**1.39**	1.01	1.90	26	**1.56**	1.01	2.40	24	1.07	0.68	1.66
**Ability to appeal**																
Can appeal	170	**1**			289	**1**			225	**1**			346	**1**		
Can appeal with help	53	0.86	0.63	1.18	110	0.93	0.74	1.17	44	**0.70**	0.50	0.98	90	0.96	0.75	1.22
Can not appeal/no help	22	**1.59**	1.01	2.51	40	1.26	0.88	1.81	23	**1.67**	1.07	2.62	33	**1.77**	1.21	2.57
**Membership in a work**																
**related association**																
Yes	162	**1**			326	**1**			231	**1**			402	**1**		
No	88	1.29	0.99	1.70	115	0.84	0.67	1.06	63	0.97	0.73	1.30	68	**0.63**	0.48	0.84
**Musculoskeletal diagnoses**	138				363				633				1072			
**Societal participation**																
**Participation in political**																
**discussions**																
Usually take part	63	**1**			115	**1**			266	**1**			370	**1**		
Sometimes take part	32	0.73	0.48	1.12	113	1.14	0.87	1.48	184	0.88	0.72	1.06	367	1.05	0.90	1.21
I usually listen, not take part	24	1.16	0.71	1.89	81	1.33	0.99	1.78	119	1.12	0.90	1.40	247	**1.18**	1.00	1.40
I do not listen or take part	18	1.44	0.83	2.49	53	**1.65**	1.17	2.33	58	**1.52**	1.13	2.03	82	**1.38**	1.08	1.76
**Ability to appeal**																
Can appeal	86	**1**			226	**1**			405	**1**			675	**1**		
Can appeal with help	41	1.40	0.96	2.06	102	1.04	0.82	1.32	176	**1.24**	1.03	1.49	320	**1.19**	1.04	1.37
Can not appeal/no help	11	**2.12**	1.12	4.03	33	1.46	0.99	2.15	44	**1.46**	1.06	2.03	72	**1.45**	1.13	1.87
**Membership in a work**																
**related association**																
Yes	93	**1**			293	**1**			535	**1**			952	**1**		
No	45	**1.48**	1.02	2.16	69	**0.60**	0.45	0.80	97	0.80	0.64	1.00	119	**0.53**	0.43	0.65

^a^ Number of cases (n).

^b^ Hazard ratio (HR) and 95% confidence interval (CI), adjusted for socio-demographic conditions, self-reported long-standing illness, age at interview, and year of interview.

Data is register information that originates from Statistics Sweden and the Social Security Authority. The data collection from Statistics Sweden is based on informed consent to answer SSLC surveys between 1990-2007. Data about granted DP originates from the Social Security Authority (MiDAS) that was collected for research purposes without consent from the individual. The Swedish law on Research Ethics states that research use of register data has to be given an approval from a Regional Research Ethics committee. The study was approved by the regional research ethics committee in 2011 Stockholm, Sweden (Dnr: 2011/1689-31/5).

## Results

The relations between the potential confounders and risk of DP are presented in Table 2. Increased risks of DP were found among foreign born individuals, with the highest estimate among younger men. Low levels of education were clearly associated with DP, and younger men and women showed higher risk estimates (HRs) compared to the older groups. For work status, an increased risk of DP was seen for those who were not employed, i.e. homemakers, job seekers or unclassified, with the highest HRs among younger men and women. As expected, having a longstanding illness, and reporting a poor SRH, was strongly related to the risk of future DP in all strata, with slightly higher HRs among the younger men and women. We decided to control for all the variables considered as confounders in the analyses of social isolation and societal participation and risk of DP (only longstanding illness was included as a measure of health status). 

### Social isolation and risk of DP

Regarding family status, the risks of DP were increased among men and women living alone (Table 3). Lack of children seemed to decrease rather than increase the risk among the younger men and women living alone, which was also seen among cohabiting younger women. For older men and women, lack of children showed somewhat higher HRs compared to those for living with children in the home. The pattern for family status remained after control for socio demographic conditions and longstanding illness, although most estimates were reduced (Table 3).

Having sparse contacts with others was related to an increased risk of DP only among younger women. Otherwise, few contacts with others showed a decrease rather than an increase in risk of future DP. After control for potential confounding the results were somewhat weakened, but the patterns remained. A similar outcome was seen for not having a close friend, i.e. a clear effect among younger women, which also remained in the multivariate analysis. 

### Societal participation and risk of DP

Individuals who did not take part in political discussions, and persons who reported that they could not appeal a decision made by a public agency, showed increased risks of DP in all strata. However, these effects were weakened after control for socio demographic conditions and longstanding illness (Table 3).

The results for not being a member of any work related association indicated an increased risk of DP among younger men. For the other strata, the risk estimates were close to unity, or showed an association in the opposite direction. After control for potential confounding, the decreased risks among both younger and older women were reinforced. 

### Social isolation and risk of DP with mental or musculoskeletal diagnosis

The results for social isolation and risk of DP were mainly attributed to DP with mental diagnoses (below referred to as mental DP). Comparatively few risk estimates differed from unity in the analyses of DP with musculoskeletal diagnoses. In the analyses of the specific diagnostic groups, the statistical precision was weakened compared with the analyses of all DP (Table 4).

Living without a partner increased the risk of mental DP. Living alone and with no children showed the highest HRs, with the exception of younger women. For DP with mental diagnoses, the patterns within the younger age groups of higher risk estimates among those with children compared to those without children remained only among young women. As for all DP, the older men and women who lived without children had an increased risk of DP with mental diagnoses. Family status was unrelated to risk of DP with musculoskeletal diagnoses among the younger men and women, but among the older groups the pattern of increased risks among those without children was evident also for DP with musculoskeletal diagnoses.

Sparse contacts with others increased the risk of mental DP only among younger women, and this was found for both mental DP and DP with musculoskeletal diagnoses. The reverse relations between contact frequency and risk of DP mainly among older men were also found for risk of mental DP. Among younger men and women with no close friends there was a clear association with mental DP, while lack of a close friend did not affect the risk of DP with musculoskeletal diagnoses.

### Societal participation and risk of DP with mental or musculoskeletal diagnosis

The results of the multivariate analysis of societal participation in relation to mental DP and DP with musculoskeletal diagnoses are shown in Table 5. For all strata except older women, those who said that they do not listen or take part in political discussions showed an increased risk of mental DP. In the analyses of DP with musculoskeletal diagnoses a similar outcome was found.

Being unable to appeal to decisions made by public authorities was associated with increased HRs of mental DP among men and older women. Also for DP with musculoskeletal diagnoses the risk estimates were elevated, with the highest HR (a doubled risk) among young men. 

The risk of mental DP was unrelated to lack of membership in a work related association, except for older women who showed a decreased risk for not being a member. However, young men who were not members showed and increased risk of DP with musculoskeletal diagnoses, while women showed an opposite association. 

## Discussion

This population based prospective study showed that social isolation and low societal participation predicted DP, particularly among younger individuals, age 20-39 years at the exposure assessment. Social isolation and low societal participation among men and women predicted DP even after socio demographic conditions and self reported longstanding illness of the individuals were taken into account. 

In the present study the risk of DP was increased among lone men and women, among older men and women without children, among women who reported that they had sparse contacts with others, or no close friend. Not taking part in political discussions indicated increased risks in all strata. The results for persons without ability to appeal a decision made by a government agency, or who were not members of a work related association showed particularly high HRs among younger men. The results for not being a member of a work related association went in the other direction among women, lack of membership decreased the risk of DP. 

These results are in line with previous studies showing associations between loneliness and weak social networks and risk of DP [33,38]. In a previous study focusing on young women, we also found that lone young mothers had an increased risk of DP [25] and that lack of employment and poor social networks, were predictors for being granted a DP among young women [24]. A number of recent studies have also shown that SRH [33,39,40], socio demographic factors including occupational class [34], and socioeconomic status [33,35,41], job strain [42,43], and psychological distress [44] can affect the risk of receiving a DP. 

The most common diagnostic group among those who participated in the present study was musculoskeletal diagnoses, followed by mental diagnoses. The results of the separate analyses for individuals who were granted DP with mental diagnoses and musculoskeletal diagnoses, respectively, showed that the negative effects of social isolation were particularly marked for those with mental DP. Low societal participation had a negative effect mainly on the risk of DP with musculoskeletal diagnoses, but also on the risk of mental DP. 

Loneliness is a qualitative, subjective evaluation related to individuals’ expectations of and satisfaction with the frequency and closeness of contacts [45,46]. The result from research on the role of social isolation and loneliness shows that both are related to illness and mortality and mainly independent even if the results are not completely consistent on this point [46,47]. Most of these studies seem to have been carried out on older people.

Exit from working life due to ill health and DP is a negative outcome for the individual and a social and economic problem for the society [1,12]. Increased social isolation, weak private networks, and low degree of participation in the society may reinforce the individual’s mental ill-health by cutting off the individual from information, communication, and relations to other people [1]. To break the vicious circle of social isolation and low societal participation of younger individuals, efforts should be made to strengthen psychiatric health care, improve possibilities for the individual to be part of social networks linked to e.g. education, social and cultural activities as well as being part of working life.

### Strengths, limitations and future research

The major strengths of the present study included the prospective design, the population based samples, and that registry data were complemented by data obtained from personal interviews. The fact that all exposures and confounders were measured at least one year ahead of the outcome reduces some of the problems related to causal inference. All individuals who had been granted DP prior to the interview were excluded. Additionally the control for self-reported longstanding illness at interview also reduced the problem that social isolation and low societal participation may have been caused by the illness that subsequently lead to DP. The study also allowed control for a number of potential confounding factors including socio demographic conditions and year at interview. 

The number of interviews was large and based on representative samples with satisfactory response rates. The specific DP diagnoses were obtained from high quality national registers, and there were only 203 out of 5724 cases without a specified diagnosis. The classification was based on primary diagnosis, which means that for an individual classified with a mental diagnosis as the primary cause, a musculoskeletal diagnosis could be recorded as well but as a co-morbidity of lower significance, and vice versa [19]. The overlapping should mainly lead to underestimated differences between the two diagnostic groups studied.

The items measuring “social isolation”, were focused on objective and quantifiable aspects. However, the question “Do you have one or more really good friends with whom you can get in contact and discuss all sorts of things?” cannot be seen as a pure indicator of being alone but can also reflect psychological aspects. “Not having close friends” may not necessarily mean enforced social isolation for all respondents. In further research the distinction between social isolation and loneliness should be developed to include a measurement of the degree of perceived absence of opportunities.

The follow-up period varied between the individuals and even if the average follow-up was 12 years it was considerably longer among individuals interviewed in the early 1990’s. A long follow-up means that important changes in life conditions may have taken place, which we were unable to take into account in this study. Median DP age was 41 and 58 years in the age groups 20-30 and 40-64, respectively, which means that the individuals labeled as “younger” received their DP on average more than fifteen years earlier in life than those labeled as “older”. 

For further research it would be of interest to differentiate between social isolation and loneliness. In such an approach social isolation is seen as an objective, quantitative measure of network size and diversity, and frequency of contact. Loneliness or perceived social isolation is believed to be its psychological counterpart. Future studies should also pay further attention to the possibility that social isolation and low societal participation may be influenced by the same illness that lead to DP.

## Conclusion

The study suggests that social isolation in relation to family and friends and low societal participation are predictors of future DP. The association found for social isolation was specifically attributed to disability pension with mental diagnoses, and to men and women under the age of 40.
